# Hepatitis E Virus Outbreak among Tigray War Refugees from Ethiopia, Sudan

**DOI:** 10.3201/eid2902.221796

**Published:** 2023-02

**Authors:** Ayman Ahmed, Yousif Ali, Nouh S. Mohamed, Jakob Zinsstag, Emmanuel Edwar Siddig, Amna Khairy

**Affiliations:** Sirius Training and Research Centre, Khartoum, Sudan (A. Ahmed, N.S. Mohamed);; Swiss Tropical and Public Health Institute, Allschwil, Switzerland (A. Ahmed, J. Zinsstag);; University of Basel, Basel, Switzerland (A. Ahmed, J. Zinsstag);; University of Khartoum, Khartoum (A. Ahmed, E.E. Siddig);; Sudan Federal Ministry of Health, Khartoum (Y. Ali, A. Khairy);; Erasmus MC University Medical Center, Rotterdam, the Netherlands, (E.E. Siddig)

**Keywords:** Hepatitis E virus, HEV, vaccine, displaced populations, virus genotype, outbreaks, zoonoses, viruses, One Health, refugees, Ethiopia, Sudan

**In Response:** We appreciate the insightful comments from Azman et al. on use of licensed hepatitis E virus (HEV) vaccine (Hecolin; Wantai BioPharm, https://www.ystwt.cn) for outbreak control regardless of virus genotype (*1*). We share their concern about the need for timely use of effective outbreak control measures, particularly among those at high risk for illness and death, such as forcibly displaced populations in humanitarian camps. We also agree that vaccines are an effective tool for prevention and control of outbreaks, including HEV (*2*).

When we confirmed an HEV outbreak among refugees from Ethiopia in east Sudan, according to the World Health Organization (WHO) recommendation, the National Immunization Technical Advisory Groups of Sudan convened an emergency meeting to discuss the feasibility of deploying HEV vaccine. After considering the WHO position paper about the use of HEV vaccine (*3*) and careful discussion, they raised several concerns about introducing the vaccine. These concerns included the limited evidence on efficacy and safety data in pregnant women, persons <16 years of age, the elderly (>65 years of age), and persons with underlying diseases (e.g., liver disease) or conditions such as immunosuppression (*3*). Of particular concern were children and pregnant women in humanitarian crisis, who are most at risk during HEV outbreaks (*4*). A major concern was that, according to the WHO statement on the vaccine, “there are no data on specific protection afforded by the HEV 239 vaccine against genotype 1, 2, or 3 HEV infection.” Therefore, the final recommendation was to invest more resources toward improving water, sanitation, and hygiene interventions until further evidence is available. In addition, several diseases prevalent among displaced populations in Sudan are preventable through water, sanitation, and hygiene interventions (*5*). These infections include waterborne diseases such as HEV and cholera (*2*,*6*); vectorborne diseases such as Chikungunya, dengue, and Rift Valley fever (*5*,*7*); and measles, meningitis, and poliomyelitis (*5*).
